# Fatigue Index and Fatigue Rate during an Anaerobic Performance under Hypohydrations

**DOI:** 10.1371/journal.pone.0077290

**Published:** 2013-10-30

**Authors:** Mohamed Nashrudin Naharudin, Ashril Yusof

**Affiliations:** Sports Centre, University of Malaya, Kuala Lumpur, Malaysia; Universidad Europea de Madrid, Spain

## Abstract

**Background:**

Since hypohydration commonly occurs in sports, studies on anaerobic exercise performance under this condition have been extensively carried out. When describing anaerobic performance, authors usually refer to a drop in anaerobic performance as fatigue index (FI) which is conventionally calculated using peak and low power data points. Meanwhile, another possible method in explaining anaerobic fatigue is using the rate constant which is derived from the exponential decline of power output known as fatigue rate (FR). Few studies have demonstrated that there was no change in anaerobic performance under mild hypohydrations.

**Purpose:**

This study aimed to compare the kinetics of power output using FI and FR of an anaerobic performance (Wingate test) under 2, 3 and 4% state of hypohydrations.

**Method:**

Thirty two collegiate cyclists (age  = 22±2 years; body weight  = 71.45±3.43 kg; height  = 173.23±0.04 cm) were matched using their baseline anaerobic peak power (APP) then randomly divided into 4 groups of EU (euhydrated), 2H, 3H and 4H respectively.

**Results:**

As expected the, FI, APP, anaerobic lower power (ALP) and rating of perceived exertion (RPE) did not show significant differences between and within the groups. However, the FR in 3H (0.018±0.005s^−1^) and 4H (0.019±0.010s^−1^) were significantly lower than EU (0.033±0.012s^−1^). Post-test FR also showed significant reduction in 3H and 4H compared to their pre-test values (p<0.05).

**Conclusion:**

Despite the lack of changes in APP and RPE, subjects in 3H and 4H showed evidence of lower reduction of power output over time. The findings support earlier reports which showed no change in anaerobic performance under mild hypohydrations. The relatively lower FR suggests higher drive in maintaining power output under hypohydrations of 3 and 4% body weight.

## Introduction

Hydration status is an important issue to be taken into consideration during sports performance, and athletes are generally urged to be well hydrated prior to competitions. Investigators have studied the effect of hypohydration on anaerobic performance however, there exist discrepancies within the body of literature, some of which show improvement of performance [1], while others demonstrate no change in performance [2], [3], [4], [5], [6], or reduction in performance under states of hypohydration [7], [8], [9], [10], [11]. Strength and power are important determinants of muscle anaerobic performance. Several studies [3], [6], [12] reported no significant difference in peak torque, vertical jump height, peak lower-body power (jump squat), or peak lower-body force (assessed via isometric back squat) during a maximal isometric voluntary contraction or for time to fatigue of knee muscles between dehydration of less than 5% body weight and control. On the contrary, upper and lower body anaerobic muscular power and isometric muscular strength significantly decreased following a hyperthermic (30 min of sauna) dehydration method [5], [12], [13], [14].

Kraft et al. [6] suggested that the equivocal evidence is associated with the variation of hydration levels tested, the mode of inducing hypohydration and testing paradigms. Many have attempted to explain the impairment of muscle anaerobic capacity based on factors such as the cardiovascular strain involved in prolonged anaerobic bouts greater than 30 seconds [3], [12], muscular damage [11], and an increase in lactate concentration [3], [10]. Meanwhile, the increase in performance has been attributed to a lighter body weight to be resisted following hypohydration [1], [3]. To our knowledge, most of the studies reported that anaerobic performance was not affected by hypohydration [3], [5], [15]. The variables measured in those studies, however did not take into account rate of the decline in power output throughout the entire test period.

In measuring anaerobic fatigue during a Wingate test, the fatigue index (FI) is ordinarily applied by taking the percentage of power drop of 2 data points (peak and low power). However, another possible method of measuring fatigue during a Wingate test which has not been reported previously is by using fatigue rate (FR) [16]. Several researchers believe that the inability to maintain power output during a Wingate test may lead to an exponential decline in power throughout the entire duration of the test indicated by the rate of fatigue [17], [18]. By taking every data point (per second) between peak and low power output, the rate of exponential decay could be calculated. Since, most of the studies reported no change in anaerobic peak power (APP) and FI at hypohydration of less than 4% [3], [6], [12], it is believed that greater physiological responses in maintaining power output will occur at levels closer to or at 4% than levels lower (1–2%). By using FR, the kinetics of power decline would reflect the changes in power output throughout the Wingate test. A lower FR would mean greater body responses to maintain the power output throughout testing period, as most study reported no change in APP.

Thus, the purpose of this study is to observe the FI and FR during an anaerobic performance test under different levels of acute hypohydrations with the assumption that there would be no change in the kinetics between the states of hypohydrations.

## Materials and Methods

### Ethics Statement

Subjects signed a consent form and received briefing on the hydration procedures and test protocols without being given too much detail about the experiment. The study was approved by the Sports Centre, University of Malaya Research Committee.

The descriptive statistics on the demographic characteristics (age, height and BMI) pertaining to all of the subjects are presented in Table 1. The fact that there was no significant difference between the groups indicated that each group was similarly characterized.

**Table 1:** Descriptive and demographic characteristic for all participants.

Subject's characteristic	EU	2H	3H	4H
**Number of subjects (n)**	8	8	8	8
**Age (year)**	22±2	23±1	22±1	23±2
**Body Weight (kg)**	70.7±4.2	68.6±11.2	66.83±6.10	67.88±7.97
**Height (m)**	1.78±0.08	1.72±0.07	1.68±0.04	1.72±0.06
**BMI (kg/m^2^)**	24±4	24±1	24±2	24±2

Legend: Values in this table are expressed as mean ± standard deviation. No significant differences were found between groups in all demographic variables. This indicates that each group was similar in character.

### Subjects

We selected thirty two healthy non-smoking male college cyclists (n = 32) who had been involved in at least 3 training sessions (a combination of anaerobic and aerobic) per week for the previous 6 months. Athletes with a mean age of 22±2 years, body weight, 71.45±3.43 kg and height, 173.23±0.04 cm participated in this study. The subjects were among varsity athletes who had never been trained at either high altitudes (low oxygen) or in hot and humid environments. The subjects were matched according to their APP, whereupon they were randomly divided into the 4 groups namely EU (the control group/euhydrated), 2H (2% of hypohydration), 3H (3% of hypohydration) 3H, and 4H (4% of hypohydration).

### Experimental Design

Hydration levels served as the independent variables, while anaerobic power performance and anaerobic kinetics of fatigue were the dependent variables. A pre and post-tests research design had been implemented for each group in this study. In order to measure subjects' anaerobic performance (FI and FR), the 30 second Wingate test was used. A week before the pre-test, a brief explanation of the experiment process and several practice sessions of the exercise protocol were given to all subjects (3 to 4 times).

During the pre-test, subjects were asked to consume 2.4 liters of water to reach a state of euhydration (divided into 1.2 liters in the morning, and 1.2 liters several hours before the exercise testing started). While during the post-test on the following day, a similar practice was implemented. Conversely, after consuming 2.4 liters of water, subjects in 2H, 3H and 4H underwent the dehydration process in the sauna. Each subject's body weight reduction after having sweated in moderate heat would indicate the percentage of hypohydration he had undergone.

In this study, in order to achieve factual effect of anaerobic performance under hypohydrations, several potential factors that may leads to inconsistency of the results have been controlled accordingly. Seeing as how the presence of hyperthermia which accompanies hypohydration also holds the potential to independently influence anaerobic exercise performance positively [14] or negatively [15], resting at least 2 hours prior to anaerobic testing following the heat exposure method is recommended [5], [10]. Each subject rested for at least 2 hours until their body temperature return to normal (37°C) at room temperature (22.7°C) with a relative humidity of 50% subsequently the heat exposure to avoid hyperthermia. The subject's core body temperature was determined using a rectal thermistor probe Model 406; Yellow Springs Instruments, Inc. To limit physiological fluctuations, subjects were also asked to abstain from performing any exercises and prohibited from consuming alcohol and caffeine for 36 hours prior to the experiment session [3], [19]. Exercises testing were conducted in an environment of standard room temperature. Similar controls existed for dietary intake during the 2 days prior to pre and post-test experimental sessions. To minimize the potential effect of reduced caloric intake on exercise performance, we encouraged subjects to consume their typical diet throughout the study.

### Experimental Procedure

#### Preliminary Procedure

Before performing anaerobic exercise testing, the subjects' hydration status was confirmed using the Body Impedance Analyzer (Tanita TBF-300A, USA), while their euhydration status was determined using a Urine Specific Gravity (USG) Refractometer (Atago, Model PAL-10S, Japan).

#### Dehydration Method

Acute body weight loss from sweating in a room with controlled heat was used as a method to estimate the subjects' level of hypohydration level in this study [20]. Prior to the process of dehydration, each subject's nude body weight in 2H, 3H and 4H groups was recorded in a state of euhydration. Once weighed, the subjects were exposed to moderate heat in a sauna set at 40°C with 20% humidity. The subjects were dehydrated in the sauna for 15 minutes [21] after which time their body weight (after having been dehydrated) was measured using weighing scales (Seca 876, Brooklyn, New York). In the event that a subject's weight had yet to reach the desired level, he would continue the process (another 15 minutes in the sauna) of dehydration until he reached 2, 3 or 4% of hypohydration accordingly. However, if a subject did not reach the targeted level of hypohydration after a period of more than 60 minutes, no further dehydration process would be allowed in order to offset any possible deleterious effects. Subjects who were unable to achieve the desired hypohydration level were asked to undergo these procedures on another day. The control group (EU) did not follow the dehydration procedures. The hypohydration level was calculated using the method of Montain et al. [21], as follows:
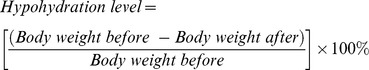



#### Wingate Anaerobic Performance Test

To measure the anaerobic capacity, each subject was required to perform a 30 second supramaximal anaerobic cycling test using the cycle ergometer (Monark 818E, Vansbro, Sweden). The ergometer was calibrated before each testing, as recommended by the manufacturer. Each subject was required to perform a pre (euhydrated) and post (hypohydrated) Wingate Anaerobic Test [22]. Test with the load resistance of the flywheel calculated based on 0.075 kg of a subject's current body weight [5]. Verbal encouragement was given during the test to ensure that the subjects performed at their maximal cycling capacity. Subject's workout intensity was measured using the Borg Scale Rating of Perceived Exertion (RPE). A scale ranging from 6 to 20 (no exertion to maximal exertion) was quantified to determine the subjects' perceived level of strenuousness after performing the testing. The subjects' heart rate (HR) responses were measured using a Polar heart rate monitor (Polar CS100 Cycling Heart Rate Monitor).

### Measures of APP ALP, TWD, FI and FR

The APP, ALP (anaerobic low power) and TWD (total work done) were calculated using Monark Anaerobic Test software (Sports Medicine Industries, Inc., St. Cloud, MN). During the anaerobic test, a sharp rise in APP was observed within the first 10 seconds of cycling [4], [5], [22], [23]. The subjects' power output showed an exponential decline in the remaining 20 seconds. To measure the level of fatiguing during the anaerobic test, the FI and FR were calculated [4], [16]. FI was determined by taking the percentage difference between maximal and minimal anaerobic performance along 30 second [24].
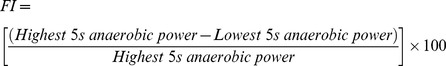



Meanwhile, FR was determined using rate constant (k) of the exponential decline of power output [4]. Similarly, the highest and lowest points of anaerobic power were established during the highest and lowest 5 seconds, respectively.
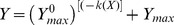
Where;
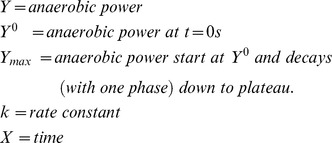



### Statistical Analyses

All statistical analysis was performed using the Statistical Package for the Social Science version 19.0′ (SPSS, Inc, Chicago, IL). A 4×2 (group × time) two-way analysis of variance (ANOVA) with repeated measure was used independently to analyze the main group (EU, 2H, 3H and 4H) and time (pre and post-test) effects on APP, ALP, TWD, FI and FR. A paired sample *t-*test was used to compare between pre and post-tests. Bonferroni post-hoc test was used to determine the significant pair wise difference. The Shapiro Wilk normality test was carried out to determine the homogeneity of the sample. The test of normality verified that all of the data produced was normally distributed (p>0.05). Statistical significance was set at p<0.05.

## Results

### APP, ALP and TWD

The means of the APP, ALP and TWD for each group are displayed in Table 2. The APP, ALP and TWD in this study elucidated that there were no significant main effects (group and time) among the 32 subjects. There were no significant differences in these three variables during the pre-test among any of the groups, with similar results obtained during the post-test.

**Table 2:** Descriptive statistics (mean ± standard deviation) for variables between groups.

	EU (n = 8)			2H (n = 8)			3H (n = 8)			4H (n = 8)		
	Pre	Post	Δ%(95% CI)	Pre	Post	Δ% (95% CI)	Pre	Post	Δ%(95% CI)	Pre	Post	Δ%(95% CI)
**Body Weight(kg)**	70.69±4.24	70.81±4.17	0.31 (0.78to −0.16)	b68.56±11.17	b67.15±10.87	−1.41 (−1.98to 0.85)	b66.83±6.10	b64.88±6.11	−1.94 (−2.32to −1.573)	b67.88±7.97	b65.06±8.08	−2.81 (−3.28to −2.35)
												
**SpecificGravity (g.m/L^−1^)**	1.012±0.004	1.012±0.004	0.001 (0.001to −0.001)	b1.012±0.003	b1.02±0.010	0.003 (−0.001to 0.007)	b1.014±0.005	b1.021±0.003	0.007 (0.001to 0.012)	b1.015±0.004	b1.022±0.004	0.007 (0.002to 0.011)
												
**AnaerobicPeak Power (W)**	618.17±197.43	620.53±183.66	7.71 (−12.28to 27.70)	610.12±209.82	614.74±156.69	17.59 (−42.02to 77.20)	613.96±118.03	587.30±124.77	−48.33 (−81.58to −15.07)	602.92±117.95	591.26±96.32	−31.11 (−80.78to 18.57)
												
**AnaerobicLow Power (W)**	365.25±87.32	355.75±87.52	−9.50 (−37.31to 18.31)	347.97±72.92	349.70±74.70	1.73 (−41.51to 44.98)	355.95±82.13	364.79±44.88	8.84 (−55.30to 72.98)	368.58±56.62	381.30±59.83	12.72 (−32.14to 57.57)
												
**FatigueIndex (%)**	78.72±6.71	79.49±7.25	−0.77 (−2.42to 0.88)	70.64±8.86	78.94±6.12	−8.29 (−29.36to 12.77)	77.30±6.10	80.30±9.24	−3.00 (−13.68to 7.69)	70.89±4.11	80.98±13.18	−10.09 (−20.56to 0.38)
												
**FatigueRate (s^−1^)**	0.029±0.017	a0.033±0.012	0.007 (−0.010to 0.020)	0.026±0.014	0.027±0.011	0.003 (−0.010to 0.030)	b0.024±0.016	b a0.018±0.005	−0.01 (−0.020to 0.010)	b 0.024±0.010	b a0.019±0.010	−0.01 (−0.010to 0.010)
												
**Total WorkDone (J)**	2514.00±556.82	2498.00±550.43	−16.00 (−156.90 to 124.90)	2443.75±474.77	2452.63±490.48	8.88 (−64.19to 81.94)	2448.63±294.80	2495.25±321.47	46.63 (−82.78to 176.00)	2468.50±268.82	2456.13±364.20	−12.38 (−160.8to 136.0)
												
**Heart Rate(beat/min)**	176±7.63	177±6.09	1.13 (−8.62to 10.87)	177±6.30	179±5.46	2.25 (−4.71 to9.21)	177±4.29	180±6.33	2.63 (−3.97 to9.22)	175±7.28	180±8.09	4.63 (−0.04to 9.29)
												
**Rating PerceivedExertion (RPE)**	17.25±0.71	16.63±0.92	−0.63 (−1.80to 0.55)	16.75±0.71	17.00±0.93	0.25 (−0.72 to1.22)	16.88±0.84	17.38±0.74	0.50 (−0.50 to1.50)	17.00±0.76	18.00±0.76	1.00 (0.01to 1.99)
												

Legend: Values are expressed as mean ± standard deviation and percentage difference of confident interval (CI).

a (p<0.05) denotes significant change between group's post-test.

b (p<0.05) denotes significant difference within group.

Body mass was significantly reduced in the dehydrated compared to the euhydrated condition.

Urine specific gravity was significantly increased (indicating dehydration) in the dehydrated compared to the euhydrated condition.

Fatigue Rate was significantly reduced in 3H and 4H compared to EU.

### Body Weight, USG, HR and RPE

All of the subjects became considerably dehydrated during the sauna due to the changes in their body weight. The body weight of the subjects was shown to have significantly decreased in 2H, 3H and 4H (p<0.05) compared to their pre-test values. Similarly, these groups also showed significant differences (p<0.05) in USG values. In HR and RPE, although there were increments in 2H, 3H and 4H, analyses showed no significant differences between and within groups.

### FI and FR

**Figure 1:**
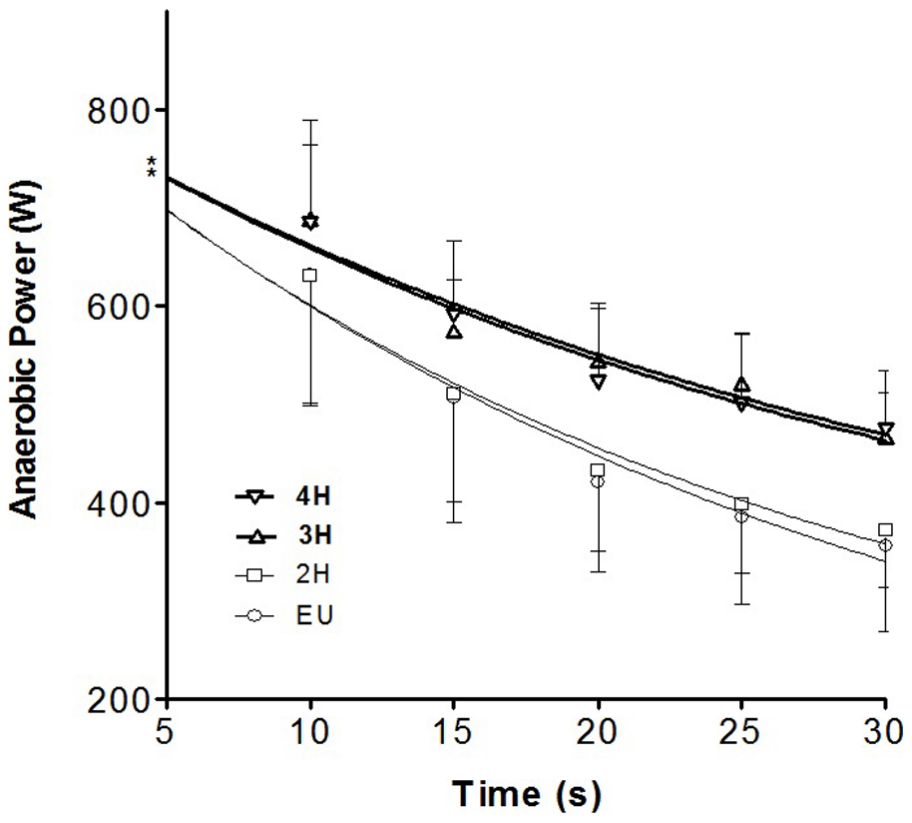
Exponential curve indicating FR for each group.

**Figure 2:**
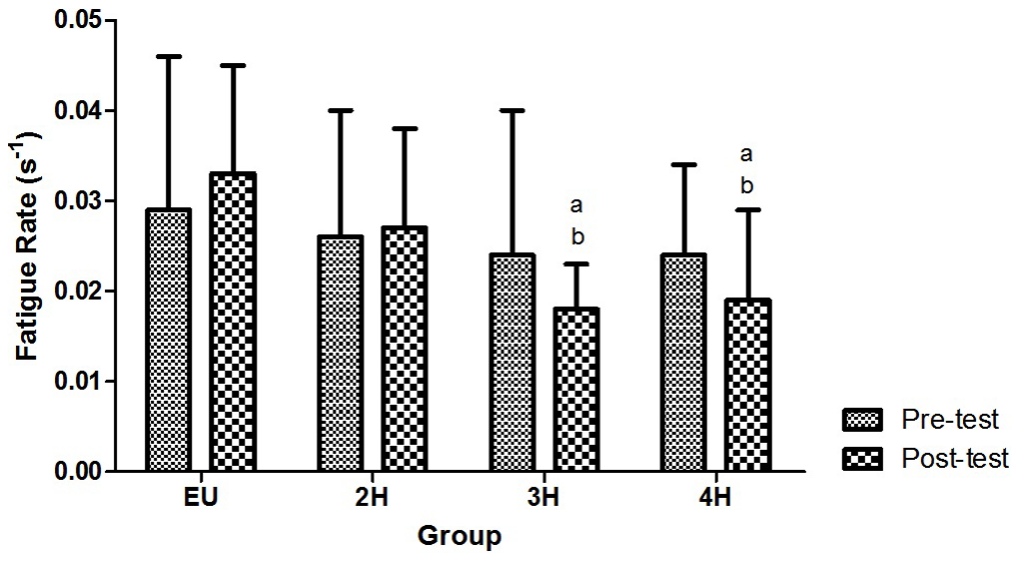
Fatigue rate pre and post test for each group.

The values of FI showed no significant effect in between and within groups in both pre and post-test. While for FR, no within group effects were observed in both pre and post-tests. However, a main effect between group of FR was observed (F_4, 30_ = 6.45; p<0.05). Although no significant differences were observed in 2H compared to the control EU group, Bonferroni post-hoc test showed a significant difference in FR between EU vs. 3H (p<0.05) and EU vs. 4H (p<0.05) as shown in Figure 1. It was also observed that FR in 3H and 4H at post-test were significantly lower (p<0.05) than pre-test (Figure 2).

## Discussion

Despite the fact that plethora of studies have examined on the effect of anaerobic performance under hypohydrations, the findings remain equivocal. Thus, it is a need for an analysis using different approaches so as to gain a more profound insight in the kinetics of anaerobic performance under hypohydration [2], [3], [6],[14]. This study was designed in attempt to observe the effect of anaerobic performance using FI and FR at different levels of hypohydrations during a Wingate test. To date there is no study on such measurements has been conducted.

Present study showed body weight reductions in 2H, 3H and 4H by 1.85±0.37%, 2.75±0.47% and 4.03±0.82% respectively, while the USG values for these groups increased significantly following the dehydration procedure. The reduction of each subject's body mass was determined by calculating the percentage from the pre-test body weight in relation to the increase in USG readings [5]. Increased perspiration produces a higher concentration of blood osmolarity, which in turn results in the kidneys acting to retain body fluid, thereby leading to an increase in urine concentration which negatively correlates with the total body water loss. Under heat exposure method of dehydration, undiminished heat may alter the muscles' metabolism, which contributes to heat exhaustion or pre-fatigue. This would add to the difficulty in determining muscle performance alone during hypohydration [21], [25], [26]. If the dehydration procedures used to reduce total body water are not sufficiently controlled, incorrectly performed, or immediately precede performance testing after the sauna exposure, confounding factors such as hyperthermia-induced muscle fatigue, neuromuscular activation deficit and metabolic changes resulted from the elevation of body temperature can affect the results [3], [21], [25], [26]. Thus, an attempt to isolate hypohydration from unwanted thermal effect after being exposed to heat in present study was successfully done. By resting for about 2 hours under room temperature prior to exercise testing, body temperature returned to baseline similar to what has been reported previously [5], [6], [26]. Consequently, it is evidence that the anaerobic peak power was unaltered after the isolation procedure of was employed. Present results strongly support most findings of earlier researchers [3], [4], [5], [6], [26] who suggested that anaerobic power performance was unaltered under mild hypohydration (2–4%). Although it is known that hypohydration leads to several physiological changes that potentially distressed exercise performance (i.e. reduced total plasma volume, increase in submaximal heart rate and decrease maximal cardiac output) [19], [21], it is believed that brief anaerobic exercise (<30 second) was independent from these changes because, it is largely relies upon stored intramuscular fuel for energy [2], [3], [19], [21], [24]. However in contrast to our findings, Jones et al. [5] reported that active dehydration of 3.1% via exercising in a hot and humid environment has a negative effect on anaerobic power. Reductions in anaerobic capacity and anaerobic power were also demonstrated among dehydrated (4.9%) wrestlers [8]. However, the active hydration implemented in those studies was different from this, where the reduction of anaerobic power could be associated with heat related fatigue and excessive workloads [3], [19].

Present study shows FI did not exhibit any changes in each of the hypohydration group and also between the groups, which means hypohydations of up to 4% did not change the gradient between APP and ALP. In other words, analyses based on FI could not discriminate the changes in power output between the groups under the range of moderate hypohydration. With the results of both APP and ALP showed insignificant differences between and within the groups, the problem has been inherently amplified that FI calculation is just based on the subtraction and division of two low-resolution values as mentioned in the methodology section [16]. Thus, the calculation of FI here might not show the actual pattern of power output throughout the testing and could be the reason behind the unchanged FI in this study.

The kinetics of power output throughout the whole period of the anaerobic test would provide relevant information on the fatiguing performance which could be invariably different from FI. Interestingly, although no significant alterations of APP and FI under hypohydrations, it is found that there were reductions of FR in 3H and 4H compared to EU. It seems that subjects in both the 3H and 4H groups were able to exert their power output (maintaining cycling cadence) better than the control group.

According to Judelson et al., [19], decrease in body mass resulted from hypohydration might offset reduced muscular power for body mass related performance. Thus, if muscular power is unaltered from hypohydration, mechanically, the working muscles will become more efficient in performing task. For example, if hypohydration fails to reduce muscle force or power as evidenced in current study (post-test APP is unchanged when compared to pre-test), number of cycling cadence actually increased as total body mass decreased because subjects resisted lesser flywheel load. In other words, if hypohydration did not reduce muscle power (APP), weight related performance (e.g. vertical jump, cycling, sprinting) should increase as total body water decreases, because the subjects are working with lesser body mass [1], [19].

Although it is not quite clear the underlying physiological mechanism, these findings may possibly be explained by the mechanism earlier proposed by De Luca et al. [27]. As the power output during the 30 second anaerobic test progressively declined in the continuously active muscle, increase excitation is required to keep the muscle output constant. The increased excitation (central drive) produces the recruitment of additional motor units. The higher activation may have influenced the rate either at the beginning of the 30 second anaerobic test [28], [29] or at the end [30]. A study by Judelson et al, [3] suggested possible differences in nervous stimulation of the musculature might occur despite no change in peak force at 2.5 to 5% of hypohydration. Although this argument is somewhat speculative, alterations in central drive seemed to be more evidenced at higher level within the moderate level hypohydration without any perceivable change in RPE.

In short, hypohydration of up to 4% does not alter APP and FI. However, the present findings indicated a significant reduction in anaerobic FR that resulted from 3 and 4% hypohydration. Hypohydration at these intensities might have produced a higher drive in maintaining the power output during the 30 seconds Wingate test. Though, it should be noted that the reductions in FR occur within very narrow parameters, and circumspection therefore needs to be exercised so as not to transgress the boundaries of these parameters, lest detrimental effects may occur as few studies have shown that anaerobic power performance was markedly reduced at hypohydration of 5% or more [8], [23], [31]. This study demonstrated the relationship between water deficit and athletes' capability to perform using anaerobic power.

It is known that intense physical activity during hypohydration is associated with various physiological changes in the human body in maintaining homeostasis. In this study, it is expected that the body will try to adapt to minimal changes in body weight during hypohydration. Adaptations were observed at 3 and 4% of hypohydrations, where the FR decreased (subject less fatigue). By calculating the FR, we are able to discriminate the differences observed between the groups; hence, we strongly suggest its use in future work involving hypohydration and anaerobic performance.

## Conclusion

Practically, it is clear that the calculation of FR provides more defined results of the anaerobic performance under narrow limits of hypohydration as compared to FI. The decreased in FR under moderate level of hypohydrations showed that the lighter body mass resulted from dehydration, makes body weight related sports such as in sprinting peak performance sustainable. Thus, this may be considered as one of sport strategy by related athletes and coaches.
